# Using Stable Isotopes to Infer the Impacts of Habitat Change on the Diets and Vertical Stratification of Frugivorous Bats in Madagascar

**DOI:** 10.1371/journal.pone.0153192

**Published:** 2016-04-20

**Authors:** Kim E. Reuter, Abigail R. Wills, Raymond W. Lee, Erik E. Cordes, Brent J. Sewall

**Affiliations:** 1 Temple University, Department of Biology, Philadelphia, Pennsylvania, United States of America; 2 Mpingo Conservation & Development Initiative, Kilwa Masoko, Tanzania; 3 Washington State University, School of Biological Sciences, Pullman, Washington, United States of America; University of Southern Denmark, DENMARK

## Abstract

Human-modified habitats are expanding rapidly; many tropical countries have highly fragmented and degraded forests. Preserving biodiversity in these areas involves protecting species–like frugivorous bats–that are important to forest regeneration. Fruit bats provide critical ecosystem services including seed dispersal, but studies of how their diets are affected by habitat change have often been rather localized. This study used stable isotope analyses (δ^15^N and δ^13^C measurement) to examine how two fruit bat species in Madagascar, *Pteropus rufus* (n = 138) and *Eidolon dupreanum* (n = 52) are impacted by habitat change across a large spatial scale. Limited data for *Rousettus madagascariensis* are also presented. Our results indicated that the three species had broadly overlapping diets. Differences in diet were nonetheless detectable between *P*. *rufus* and *E*. *dupreanum*, and these diets shifted when they co-occurred, suggesting resource partitioning across habitats and vertical strata within the canopy to avoid competition. Changes in diet were correlated with a decrease in forest cover, though at a larger spatial scale in *P*. *rufus* than in *E*. *dupreanum*. These results suggest fruit bat species exhibit differing responses to habitat change, highlight the threats fruit bats face from habitat change, and clarify the spatial scales at which conservation efforts could be implemented.

## Introduction

Anthropogenic changes to tropical forests have been extensive. More than 350 million hectares have been removed globally [1,2], changing the availability of food resources, and modifying foraging behavior in mammals [3]. In fruit bats, modifications of the foraging behavior in response to anthropogenic habitat change can have particularly important ecological consequences in and near tropical forests. Because of the key roles of fruit bats as pollinators and seed dispersers of tropical trees [4], and because some fruit bat species can use degraded forests [5, 6] and shift from native to non-native food sources [7], foraging behavior responses by fruit bats to habitat change may have a disproportionate influence on tree reproduction and regeneration in tropical landscapes [4,8,9].

Bat foraging studies typically use a standard set of methods: cafeteria trials on captive bats [10], pollen/seed sampling from captured bats [11], field examination of feeding refuse [7], guano analyses [12], direct observations of free-ranging bats [13], radio telemetry [5], and stable isotope analyses [14]. Of these methods, stable isotope analyses provides several advantages for long-term and large-scale examination of fruit bat diets and the factors that affect them. First, many fruit bats are sequential specialists, intensively focusing on one or a few resources at a time, then rapidly shifting to new resources in accordance with tree phenology [15]. While direct observational methods require a long-term data set to understand the complete annual diet of sequential specialists, stable isotope analyses can provide insight into foraging over time periods encompassing such temporal shifts (data can represent diets over several weeks or years, depending on the sample [16,17]). In addition, fruit bats travel quickly, forage over tens of kilometers, and forage in diverse habitats [18], which can be difficult to track with standard methods, yet stable isotope analyses can provide insights into bat foraging across its entire range, not only in a single location. The use of stable isotopes does have some disadvantages, including the need to obtain physical samples from bats and to assume baseline homogeneity of stable isotopes [19]. Nevertheless, when combined with a sampling regime that accounts for multiple individuals in different populations across large geographic areas, stable isotope analyses can provide new avenues for understanding how dietary composition, vertical stratification, and diet breadth shift at the population level in response to large-scale habitat change.

Previous work has demonstrated the versatility of stable isotopes for investigating shifts in fruit bat foraging behavior resulting from habitat change [14]. Stable isotopes (δ^15^N and δ^13^C) have been used in nectar-feeding bats to identify when diets switch from C3 to C4 plants in a nitrogen-limited diet (δ^15^N values are lower in C3 than C4 plants [20]), to hypothesize the presence of commercially grown fruits in diets (δ^15^N may be higher in agricultural soils due to nitrogen fertilizers [21]), and to determine the direction of seeds dispersed by bats (δ^13^C is higher in seeds in successional sites than the primary forest [22]). In addition, δ^13^C values have been used in bats to assess vertical stratification–the tendency of different species to feed at different canopy heights–among species that co-occur (δ^13^C values decrease at higher canopy strata [23]). Finally, stable isotopes can be used to examine diet breadth (a wider range of stable isotope values in a sample of bats indicates a wider diet breadth at the population level [24]).

The potential for stable isotope analyses to increase understanding of how fruit bat foraging behavior changes in response to habitat change could prove particularly beneficial in Madagascar, where more than 80% of the forest cover has been lost and most of the remaining forests are fragmented or degraded [25,26]. Madagascar’s fruit bat community is comprised of three species–*Pteropus rufus*, *Eidolon dupreanum*, and *Rousettus madagascariensis*–that are all important seed dispersers and pollinators [12,27], but that also have declining populations of conservation concern (*P*. *rufus* and *E*. *dupreanum* are Vulnerable and *R*. *madagascariensis* is Near Threatened [28]). Methods other than the use of stable isotope analyses to evaluate foraging behavior in these species have some limitations due to the diversity of habitats used by these species (including both intact and degraded forest [29,30,31,32] across broad, overlapping ranges [33]) and the large areas covered by individual bats during foraging (although these species do not migrate seasonally [34,35], they can travel up to 30 km in one night [36]). Perhaps in part because of these limitations, these species remain poorly understood [37], and no studies have compared their foraging habits across large spatial scales.

This study aimed to compare the feeding ecology of Malagasy fruit bats in light of the large-scale habitat changes occurring in Madagascar. The objectives were to examine how fruit bat diets varied (1) by species and (2) across space as a result of changes in forest cover. Due to small sample sizes, *R*. *madagascariensis* is only included in some analyses; most analyses include only *P*. *rufus* and *E*. *dupreanum*. For objective one, we hypothesized that, although all three species are largely frugivorous [12,31,38,39] their diets would differ. Specifically, in accordance with previous evidence of differences in the composition and abundance of food items in the diet of the three species [13,33], we expected among-species differences in diet would be stronger than within-species differences. Similarly, we hypothesized that diet breadth would differ between species; we expected that, in accordance with previous studies on dietary diversity, *P*. *rufus* would have the most largest diet breadth [12,31], *R*. *madagascariensis* would have an intermediate diet breadth [38], and *E*. *dupreanum* would have the smallest diet breadth [39]. We further hypothesized that the bats would exhibit vertical stratification (a type of resource partitioning) when multiple species were present in the same area [14]. Based on previous field observations [33], we expected that stable isotope values would be consistent with *P*. *rufus* feeding at higher vertical strata than *E*. *dupreanum*.

For objective two, we hypothesized that the bat diets would differ spatially. Because the extent of anthropogenic degradation of forest habitat (but not forest type) varied across our study region, we expected their diets would vary regionally. Specifically, because of the different types of fruit resources consumed by bat species in degraded areas [14,29,31], and because of the long nightly distances they can fly [18], we hypothesized that diets would change as the percent of forest cover decreased at spatial scales close to the maximum nightly flight distances.

## Methods

### Ethical Research Statement

Research design, including the recruitment of hunters and meat sellers into the study, was approved by an ethical review board (Temple University Institutional Review Board, protocol number 21414), as was the collection of hair samples from wild animals (exempted study because no live animals were involved in the research, Temple University Institutional Animal Care and Use Committee). No animals were killed specifically for this study (see Methods). Data were not collected on how animals were killed; the actual capture and killing of the animals was outside the scope of the researchers’ interactions with hunters and meat sellers. Samples were collected from carcasses at the point of sale in urban towns (e.g., where hunters sold carcasses to market vendors or other individuals; at market stands directly to the public; in restaurants); these locations were typically at least 5 km distant from the rural locations where the bats were hunted. To avoid either creating incentives promoting wild meat hunting or imposing burdens on those participating in the study, sampling was undertaken so as to provide neither cost nor benefit to meat sellers. Meat sellers were not paid for their participation, but were reimbursed for any telephone credit used for the study. One exception was for one market vendor in Antsiranana, who was reimbursed a nominal amount (USD$0.50–1.00 per sample for 17 samples; market price of one bat was USD$1.00–2.50) to compensate for a reduction in sale price of the bat meat due to removal of hair samples. In Antsiranana, Anivorano Nord, Andriba, and Ansiafabositra, hair samples were also collected from bats via hunters. These hunters were recruited through a related research project [40] and were not compensated. Research was conducted under the authorization of the Madagascar Ministry of Water and Forests. Permission to conduct research was also requested and obtained from the highest ranking, locally elected official of each town. Hair samples were exported from Madagascar and were declared to the U.S. Fish and Wildlife Service at the New York City port of entry.

### Study Site

**Fig 1:**
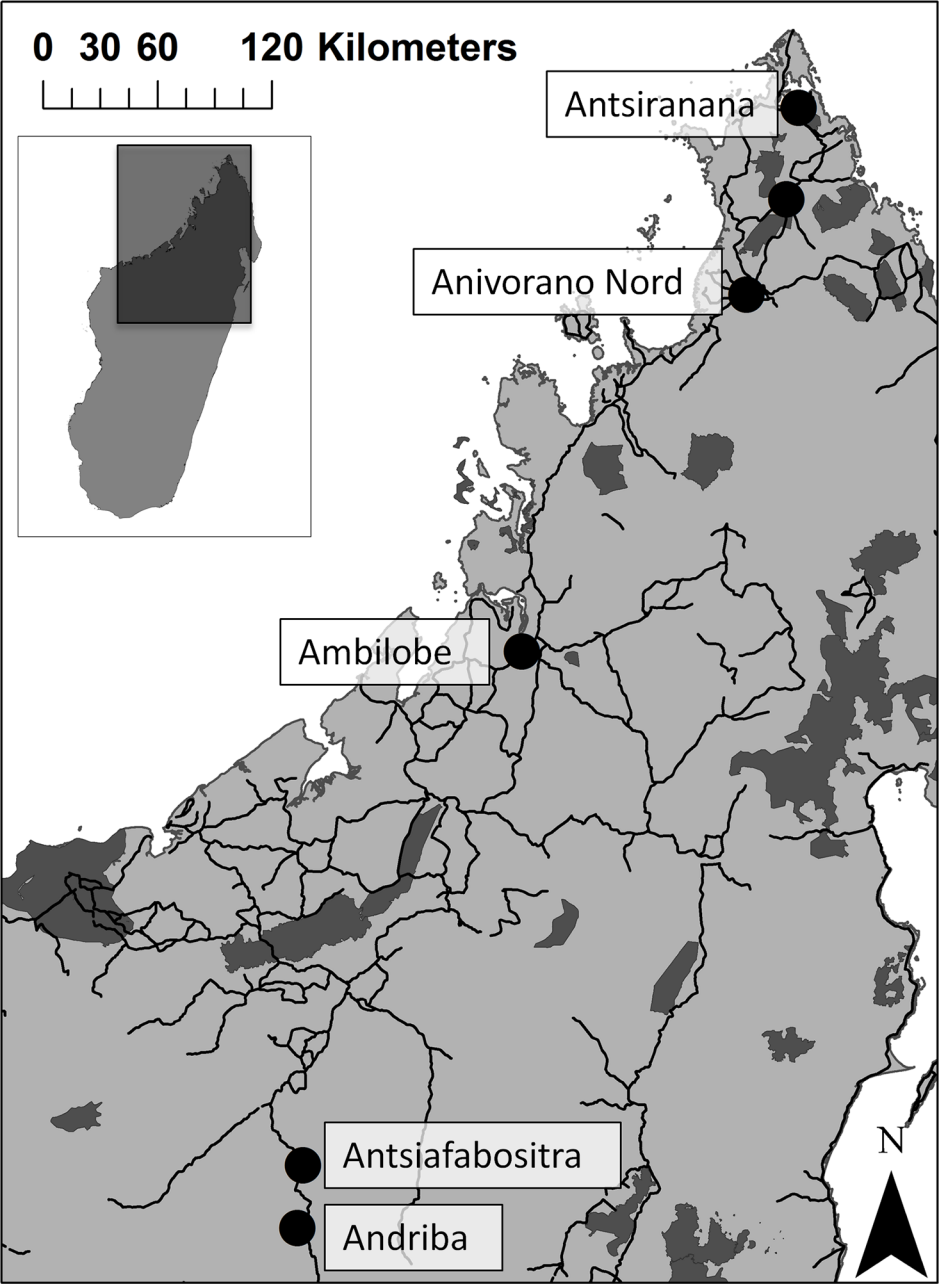
Locations of cities, indicated by black circles, where wild meat samples were collected. Dark gray regions denote protected habitat, black lines indicate roads, and the inset indicates the location of the study region on the island of Madagascar. Reprinted under a CC BY license, with permission from Reuter, original copyright 2015 [41].

Samples were collected (June-August 2013) in six towns in Madagascar where there were formal bat traders (i.e., organized, persistent, publicly known, entities involved in the regular trade of bats) (Table 1, Fig 1). The towns were 45 to 425 km apart, and varied in distance from the coast and from protected areas (Fig 1). Four of the towns were near known roosting sites for at least one of the species [32]. Given that samples were collected from formal traders in towns, they were not systematically collected across the landscape, though the traders always sourced bats from a variety of hunting locations around each town.

**Table 1:** Towns where hair samples were collected, with hunting sites for each town.

Town (hunting sites listed under each town)^a^	Number of meat sellers enrolled in study	Town population^b^	Number of individuals sampled		
			*P*. *rufus*	*E*. *dupreanum*	*R*. *madagascariensis*
*Andriba*	1	32,000			
Mangasoavina			-	1	-
*Antsiafabositra*^c^	2	8,328	4	-	-
*Antsohihy*	1	105,317			
Ambaliha			12	-	-
Ambodimanany			1	-	-
Amboroho			2	-	-
*Ambilobe*	1	56,427			
Ambakiarano			-	11	-
Amborondolo			30	-	1
Beramanja			15	-	-
Isesy			15	2	-
Mahivoragno			-	16	-
Mamoro			3	-	-
*Anivorano Nord*^d^	6	15,000	6	20	-
*Antsiranana*	4	87,569			
Akonokono			15	2	-
Andranofanjava			16	-	-
Daraina			7	-	5
French Mountain			7	-	-
Mangoaka			5	-	1
**Total:**	**15**	**-**	**138**	**52**	**7**

Towns in which data were collected along with the number of meat sellers enrolled in the study, the human population of the towns in which data were collected, and the number of hair samples collected in each town by species.

^a^Hunting sites are those reported by hunters and meat sellers in each town; this may not be an exhaustive list of hunting sites for each town.

^b^Town population estimates were taken from the Ilo database [44].

^c^In Antsiafabositra the hunting site or sites were unknown, so we do not differentiate by hunting site.

^d^In Anivorano Nord, bats were hunted only at one hunting site but the name of this hunting site is unknown.

### Bat Hair Samples

We used bat hair for stable isotope analyses (as opposed to tissues) because the slow turnover rate of hair integrates diet information over a long period (several months [16]). No bats were captured. Samples were obtained from bat carcasses from hunters or wild meat vendors including restaurants and food market stands during the legal hunting season (i.e. when bats are legal to hunt and sell in Madagascar, Table 1 [29]). The bat carcasses was always intact (e.g., not cut into pieces) with no hair burned off or otherwise damaged, though all carcasses generally showed signs of small injuries (e.g., the causes of death). All three species are sold through the wild meat trade [42]. Sampling carcasses from the wild meat trade facilitated collection of samples across a large spatial scale and has been used to collect hair and tissue samples for ecological studies in other areas of Africa [43]. This sampling procedure could have introduced some bias if hunters hunted in areas not representative of typical foraging areas or if hunters prioritized some bat species over others. Such bias is considered minimal for this study since hunters have an economic incentive to capture bats where they are most abundant, because hunters often use indiscriminate hunting techniques such as large nets that capture any species present [42], and because our study focused on the regional scale and hunter preferences for particular types of hunting grounds or species may not be consistent across large spatial scales.

Samples of ventral hair were collected from each bat carcass by a trained research team member within 24 h of bat capture. Hair was cut with sterilized scissors, then stored in labeled 1.5-mL plastic, centrifuge tubes. Care was taken to avoid contamination by blood. Hair samples were also collected opportunistically from other mammals being sold by the same meat sellers to provide the first, or some of the first, reported values of stable isotopes for those species (Table 2). The data from hair samples of these other non-bat mammal species are not analyzed further due to small sample sizes; they are presented for informational purposes only.

**Table 2:** Table of stable isotope values by mammal species, with means ± SD.

Bat Species (number of hunting sites)	δ^13^C (‰)	δ^15^N (‰)
***E*. *dupreanum* (n = 6)**		
Average	-21.33 ± 0.79	7.25 ± 0.84
Median	-21.53	7.39
Minimum Value	-19.86	5.95
Maximum Value	-21.99	8.43
***P*. *rufus* (n = 14)**		
Average	-22.44 ± 0.75	6.69 ± 0.86
Median	-22.70	6.48
Minimum Value	-23.35	5.42
Maximum Value	-21.85	8.74
***R*. *madagascariensis* (n = 3)**		
Average	-21.57 ± 0.90	8.21 ± 1.44
Median	-21.45	7.99
Minimum Value	-22.53	6.90
Maximum Value	-20.74	9.76
**Other Mammal Species**		
***Felis silvestris* (Wild Cat, n = 1)**	-20.39	11.52
***Potamochoerus larvatus* (Wild Pig/Bushpig, n = 3)**	-22.77 ± 0.61	8.81 ± 0.62
***Setifer setosus* (Greater Tenrec, n = 1)**	-21.81	10.70
***Tenrec ecaudatus* (Common Tenrec, n = 2)**	-19.12 ± 2.73	7.14 ± 1.91
***Viverricula indica* (Small Indian Civet, n = 4)**	-18.96 ± 0.95	9.09 ± 0.78

Means and standard deviations are calculated using hunting sites are replicates for bats and individuals are replicates for other species. Minimum and maximum values depict the hunting site with the lowest mean value and the highest mean value.

### Stable Isotope Analyses

We used both δ^13^C and δ^15^N stable isotopes in this study, because of the known relationship of these isotopes with foragers’ diet [14]. While δ^13^C may also reflect the photosynthetic pathway of food plants or vertical differences in foraging, most plants used for food by fruit bats in the region we studied likely use the C3 pathway, and in the seasonally dry forest region we studied, tree height is restricted (and thus vertical differences in canopy height differences are limited). Further, while δ^15^N may also indicate the trophic level of the forager, the presence of legumes, or the agricultural application of fertilizers to food crops, the focal species all forage on plants at the same trophic level (i.e., plants [18]), and fertilizer application to fruiting trees on typical small-scale farms in Madagascar is limited (BJS pers. obs.). Finally, although δ^15^N isotopes can vary considerably in dry deciduous forests [45], we argue that, as described in the ‘Statistical Analysis’ section below, geography and vegetation type both likely play a limited role in influencing isotope values in this dataset. We therefore interpret variation in δ^13^C and δ^15^N isotopes as primarily indicating dietary differences, and only secondarily vertical stratification (δ^13^C differences) or foraging in human-modified landscapes (δ^15^N). The use of δ^13^C and δ^15^N isotopes also facilitated comparison against previous studies on the same species [14].

Hair samples (0.3–0.7 mg) were washed in ethanol and packaged in tin capsules for mass spectrometry [46]. Samples were analyzed using a Costech elemental analyzer interfaced with a continuous flow Micromass (Manchester, UK) Isoprime isotope ratio mass spectrometer (EA-IRMS) for ^15^N/^14^N and ^13^C/^12^C ratios [46]. Measurements are reported in δ notation and were calculated using the following formulae:

$$\delta^{13}\text{C}=\left(\left({}^{13}\text{C}/{}^{12}\text{C of sample}\right)/\left({}^{13}\text{C}/{}^{12}\text{C of Peedee Belemnite}\right)-1\right)\;\text{x}\;1000$$

And:

$$\delta^{15}\text{N}=\left(\left({}^{15}\text{N}/{}^{14}\text{Nof sample}\right)/\left({}^{15}\text{N}/{}^{14}\text{N of atmospheric nitrogen}\right)-1\right)\;\text{x}\;1000$$

Ovalbumin was used as a routine standard (2 standards for each set of 10–12 samples). Precision for δ^13^C and δ^15^N was generally ± 0.2‰ and ± 0.4‰ (standard deviation of 10 laboratory standards). The stable isotope values (δ^13^C and δ^15^N) for each hair sample can be found in S1 Appendix.

### Social Surveys

Hunters and meat sellers provided information about the location each bat was hunted (‘hunting site’). Meat sellers sometimes sourced bats from more than one hunting site (Table 1). GPS coordinates for hunting sites were recorded during visits to hunting sites with hunters, or by extracting coordinates from maps or satellite images on the basis of landmarks indicated by the hunter or meat seller. GPS coordinates can be found in S1 Appendix.

### Statistical Analyses

Unless otherwise noted, tests were completed with JMP statistical software (JMP^®^, Version 10. SAS Institute Inc., Cary, NC, 1989–2007). Summary data are the mean ± SD. Sample sizes for *R*. *madagascariensis* were too low to include in most statistical analyses however we include data for this species when possible throughout the results because relatively little is known about this species. Because variation may be greater among than within hunting sites, hunting locations were used as replicates or random effects whenever possible. When this was not possible, we have noted the type of replicate used next to each statistical test.

Dietary differences among species were analyzed using a Permutational ANOVA (individuals as replicates) and pairwise post-hoc tests (Primer statistical software [47]) of Bray-Curtis similarity, a measure of similarity that can examines relationships among replicates in combined δ^13^C and δ^15^N values (as opposed to analyzing δ^13^C and δ^15^N values separately). This statistical test and software are frequently used to test for changes or differences in isotopic signatures (δ^13^C and δ^15^N [48,49,50]). Dietary differences and overlap were also visualized and diet breadth calculated using standard Bayesian ellipses adjusted for small sample size (SEA_c_) using the SIBER package for R statistical software [51].

Differences in dietary breadth were examined by using the Bayesian approach (10^5^ posterior draws) for comparing the standard ellipse areas of the different species [51].

We tested for vertical stratification in two ways. First, we examined whether *P*. *rufus* and *E*. *dupreanum* differed in their δ^13^C and δ^15^N values. We used mixed effects models with ‘species’ as the independent variable and a dependent variable of either δ^13^C values or δ^15^N values. A random effect [52] for ‘hunting site’ was included in each model to account for the non-independence of the isotopic signatures of bats within hunting sites. Second, we examined whether vertical stratification was evident when *P*. *rufus* and *E*. *dupreanum* were captured together (on the same night by the same hunter) versus when they were captured separately. We used a Wilcoxon Test with hunting sites as replicates to compare stable isotope values when *P*. *rufus* and *E*. *dupreanum* were captured together or separately. Four Wilcoxon tests were performed: 1) comparison of *P*. *rufus* δ^13^C values at hunting sites where no *E*. *dupreanum* were caught (n = 11) versus sites where *E*. *dupreanum* were also caught (n = 3); 2) comparison of *P*. *rufus* δ^15^N values at hunting sites where no *E*. *dupreanum* were caught (n = 11) versus sites where *E*. *dupreanum* were also caught (n = 3); 3) comparison of *E*. *dupreanum* δ^13^C values at hunting sites where no *P*. *rufus* were caught (n = 3) versus sites where *P*. *rufus* were also caught (n = 3); and 4) comparison of *E*. *dupreanum* δ^15^N values at hunting sites where no *P*. *rufus* were caught (n = 3) versus sites where *P*. *rufus* were also caught (n = 3);

We tested for regional difference in diet using Permutational ANOVA tests [47], by evaluating whether δ^13^C and δ^15^N values changed for *P*. *rufus* or *E*. *dupreanum* across hunting sites. Individuals were replicates.

For objective two, we used a model selection approach to understand how forest cover and climate affected stable isotope values. For these models, the primary predictor variable related to forest cover, which was calculated from satellite imagery [53], using images taken by the Landsat 8 satellite during August-October 2013. These dates were selected to match the time frame of hair samples collection, and because images showed low cloud cover. To evaluate the influence of the spatial scale of local habitat change on bat diets, satellite images were clipped to three different radii (5, 15, and 30 km) using ArcGIS [54]; 30 km has been estimated as the maximum foraging radius for *P*. *rufus* which has the largest nightly flight distance of the three species [18,36]. Clipped images were processed using CLASlite (version 3.1 [55]), a program used to classify tropical landscapes into forest and non-forest cover [56]. Forest cover was conservatively defined as a pixel with no more than 5% bare ground and no less than 85% live vegetation (30 meter spatial resolution [55]). Post-processing, raster files were analyzed for their percent forest cover using ArcGIS [54].

To evaluate whether changing stable isotope values could result from habitat or geographic differences across the study area, we evaluated whether dominant vegetation near hunting sites varied. Countrywide vegetation maps developed between 2003 and 2006 [57] showed that one forest type—Western Dry Forest—was the dominant non-degraded forest type within 30 km at all hunting sites (Table 3), suggesting that the role of habitat in isotope variation may be limited. To evaluate variation in stable isotope values across the study area, stable isotope values from leaves of a fruiting tree species that occurs across this region (*Mangifera indica*) were compared. While it would have been preferable to present stable isotope data from additional sites and species, difficulties in reaching hunting sites and time and resource limitations on sampling precluded broader collection of reference samples. Sample sizes were too small, however, to determine whether δ^13^C and δ^15^N stable isotope values in *M*. *indica* changed across the study area (but the values are presented in Table 4 for reference purposes). However, because all of the bats came from the same habitat type (Western Dry Forest), we suggest that the role of habitat in isotope variation is limited. Thus, in this study, we suggest that the observed differences in isotope values are primarily indicative of dietary differences. We acknowledge the limitations of this assumption.

**Table 3:** Annual Temperature Range and forest cover characteristics of each hunting site.

Town (hunting sites listed under each town)	Annual Temperature Range (Mean)^a^	Percent forest cover (radius)^b^ in 2013			Percent of native remnant primary vegetation by forest type (30 km radius) in 2003^e^			
		5km	15km	30km	WDF	W	HF	M
*Andriba*								
Mangasoavina	190	13%	18%	27%	59%	0%	41%	0%
*Antsiafabositra*^c^	183	11%	11%	4%	90%	0%	<1%	0%
*Antsohihy*								
Ambaliha	146	25%	24%	11%	68%	4%	6%	23%
Amboroho	172	6%	2%	5%	69%	3%	5%	24%
*Ambilobe*								
Ambakiarano	148	19%	1%	5%	96%	2%	2%	<1%
Amborondolo	155	53%	56%	24%	74%	5%	1%	19%
Beramanja	149	33%	30%	35%	55%	10%	<1%	35%
Isesy	146	6%	14%	5%	67%	12%	<1%	21%
Mahivoragno	135	2%	3%	5%	75%	10%	1%	14%
Mamoro	144	23%	25%	40%	66%	5%	10%	20%
*Anivorano Nord*^d^	132	48%	7%	11%	70%	<1%	29%	0%
*Antsiranana*								
Akonokono	119	19%	6%	6%	73%	10%	10%	6%
Andranofanjava	125	53%	5%	11%	48%	5%	44%	2%
Daraina	122	51%	54%	27%	55%	4%	34%	7%
Mangoaka	113	5%	13%	7%	60%	10%	26%	4%

Characteristics of the hunting sites, including: annual temperature range (mean), percent of remaining forest cover within 5, 15, and 30 km of the hunting side, and percent of native vegetation by forest type.

WDF = Western Dry Forest/Deciduous Seasonally Dry Forest. W = Wetlands/Marshlands. HF = Humid Forest/Evergreen Forests. M = Mangroves.

^a^Data derived from WorldClim database; this database expresses temperature as degrees Celsius multiplied by 10 [58].

^b^Data extracted from satellite images downloaded from the USGS Earth Explorer database [53].

^c^It was not possible to determine where the bats were hunted so the center of the town was used as the ‘hunting site’.

^d^Bats were hunted only at one hunting site, but the name of this hunting site is unknown.

^e^Data derived from Moat and Smith [57].

**Table 4:** Table of stable isotope values for the leaves of *Mangifera indica* (Common name: Mango).

Town	δ^13^C (‰)	δ^15^N (‰)
Antsiranana (n = 2)		
Mean ± SD	-29.64 ± 0.72	3.92 ± 1.12
Median	-28.61	4.48
IQR	1.43	2.23
Anivorano Nord (n = 3)		
Mean ± SD	-30.11 ± 0.71	3.63 ± 1.53
Median	-30.41	3.50
IQR	1.32	3.07
Ambilobe (n = 3)		
Mean ± SD	28.61 ± 0.09	4.47 ± 1.66
Median	-29.76	4.13
IQR	1.43	2.23
Antsohihy (n = 5)		
Mean ± SD	30.10 ± 0.88	3.78 ± 2.56
Median	-29.81	2.87
IQR	1.37	4.32

Stable isotope values (individuals are replicates) at sites spread across a distance of 440 km. Samples were collected within 30 km of the towns; leaves were collected directly from trees and were dried prior to storage in sealed plastic containers. Samples (100 mg per tree) were analyzed using the same procedures as for the hair analysis. IQR = Interquartile range.

To control for the effect of climate on stable isotope values [59], we also examined the annual temperature range of each site. This variable was used as a proxy for local climate due to its strong correlation with other climate variables (annual precipitation levels, precipitation seasonality, temperature seasonality; P < 0.05 and |*r*| > 0.8 for all pairwise comparisons at hunting sites). Annual temperature range was calculated by clipping the WorldClim [58] raster file of temperature data from 1950–2000 to a 30 km radius of each hunting site, and averaging across all pixels included within the clipped radius. Forest cover and climate data are presented in Table 3.

The importance of forest cover on bat diets was analyzed using an information theoretical approach employing model estimation and selection [60,61]. For *P*. *rufus*, a set of candidate mixed effects models was established a priori and all met the assumptions of linear mixed models. Candidate models included forest cover in %, annual temperature range, and interactions between these two variables (S1 Table). Models were validated by calculating the R^2^ of the best-fit models and these values are reported in the results. Large sample sizes that allowed for the use of individual bats as replicates, and hunting site was used as a random effect. However, because of small sample sizes in *E*. *dupreanum*, only regression models with one predictor variable were considered, and hunting sites were treated as replicates. To facilitate comparison against *E*. *dupreanum*, regression models for *P*. *rufus* were also evaluated separately using hunting sites as replicates. The full set of these candidate regression models for *E*. *dupreanum* and *P*. *rufus* are also in S1 Table. Models were ranked separately for each species on the basis of the corrected Akaike’s Information Criterion (AIC_c_), which is adjusted for small sample size [60]. Delta AICc (ΔAIC_c_) and Akaike weights (*w*_*i*_) were calculated for comparison purposes [61]. The best model was the model with the lowest AIC_c_; given the conceptual similarity of candidate models only others with substantial support were considered (ΔAIC_c_ < 2). The mixed effects models in *P*. *rufus* and regression models in *P*. *rufus* and *E*. *dupreanum* with the most support (the lowest ΔAIC_c_) were further examined to determine the relative influence of the forest cover in % and annual temperature range variables on the δ^13^C and δ^15^N values.

## Results

### Sampling Effort

Hair samples from fruit bats (n = 138 for *P*. *rufus*, n = 52 for *E*. *dupreanum*, and n = 7 for *R*. *madagascariensis*) were taken from six towns and linked via social surveys to 17 hunting sites (Table 1). Stable isotope data from opportunistic samples of wild cat (*Felis silvestris*), wild pig (*Potamochoerus larvatus*), greater tenrec (*Setifer setosus*), common tenrec (*Tenrec ecaudatus*), and small Indian civet (*Viverricula indica*) were collected opportunistically; results are provided in Table 2.

### Species Differences in Diet, Diet Breadth, and Resource Partitioning

#### Diets

**Fig 2:**
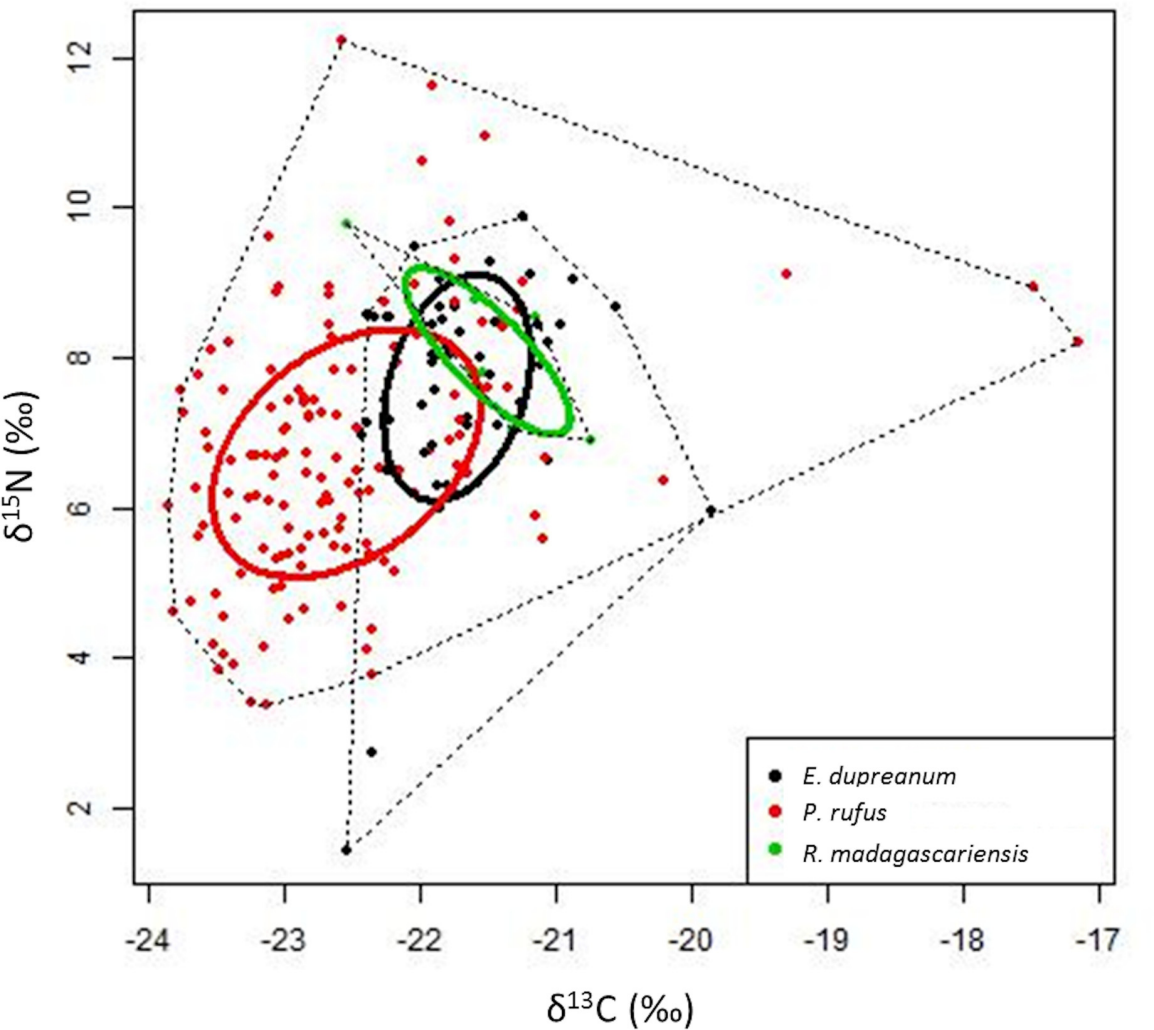
Dietary differences and overlap by species. δ^13^C and δ^15^N values of individual bats with species differences and overlaps in diet visualized using standard ellipses adjusted for small sample size (colored circles) and convex hulls (dotted lines) [51]. Standard ellipses calculated to include 40% of the data points within a species. Convex hulls capture all data points within a species.

In accordance with our hypothesis, diets (Bray-Curtis similarity of δ^13^C and δ^15^N values) differed among species (PERMANOVA, Pseudo-F = 12.99, df = 2, p = 0.001; individuals as replicates), with *P*. *rufus* having different stable isotope values than both *E*. *dupreanum* (Pairwise PERMANOVA, t = 4.63, p = 0.001) and *R*. *madagascariensis* (Pairwise PERMANOVA, t = 2.43, p = 0.015). *E*. *dupreanum* had the highest δ^13^C values while *R*. *madagascariensis* had the highest δ^15^N values (Fig 2).

#### Diet breadth

The diet breadth (calculated as the standard ellipse area or SEA_c_) was 4.75 for *P*. *rufus*, 2.45 for *E*. *dupreanum*, and 1.26 for *R*. *madagascariensis* (Fig 2). In accordance with our hypothesis, the diet breadth of *P*. *rufus* was larger than the diet breadths of *E*. *dupreanum* (comparison of 10^5^ posterior draws, p < 0.01) and *R*. *madagascariensis* (p = 0.027). There was, however, no difference in the diet breadths of *E*. *dupreanum* and *R*. *madagascariensis* (p = 0.32; S1 Fig).

#### Vertical stratification

Vertical stratification was apparent in two lines of evidence. First, when compared across all hunting sites, *P*. *rufus* had lower δ^13^C values than *E*. *dupreanum* (Mixed Effects Model, Estimate = 0.39, Std. Error = 0.12, F-Ratio = 11.84, P = 0.0008, hunting site as a random effect). However, the two species did not differ in their δ^15^N values (Mixed Effects Model, Estimate = 0.38, Std. Error = 0.20, F-Ratio = 3.60, P = 0.06, hunting site as a random effect).

**Fig 3:**
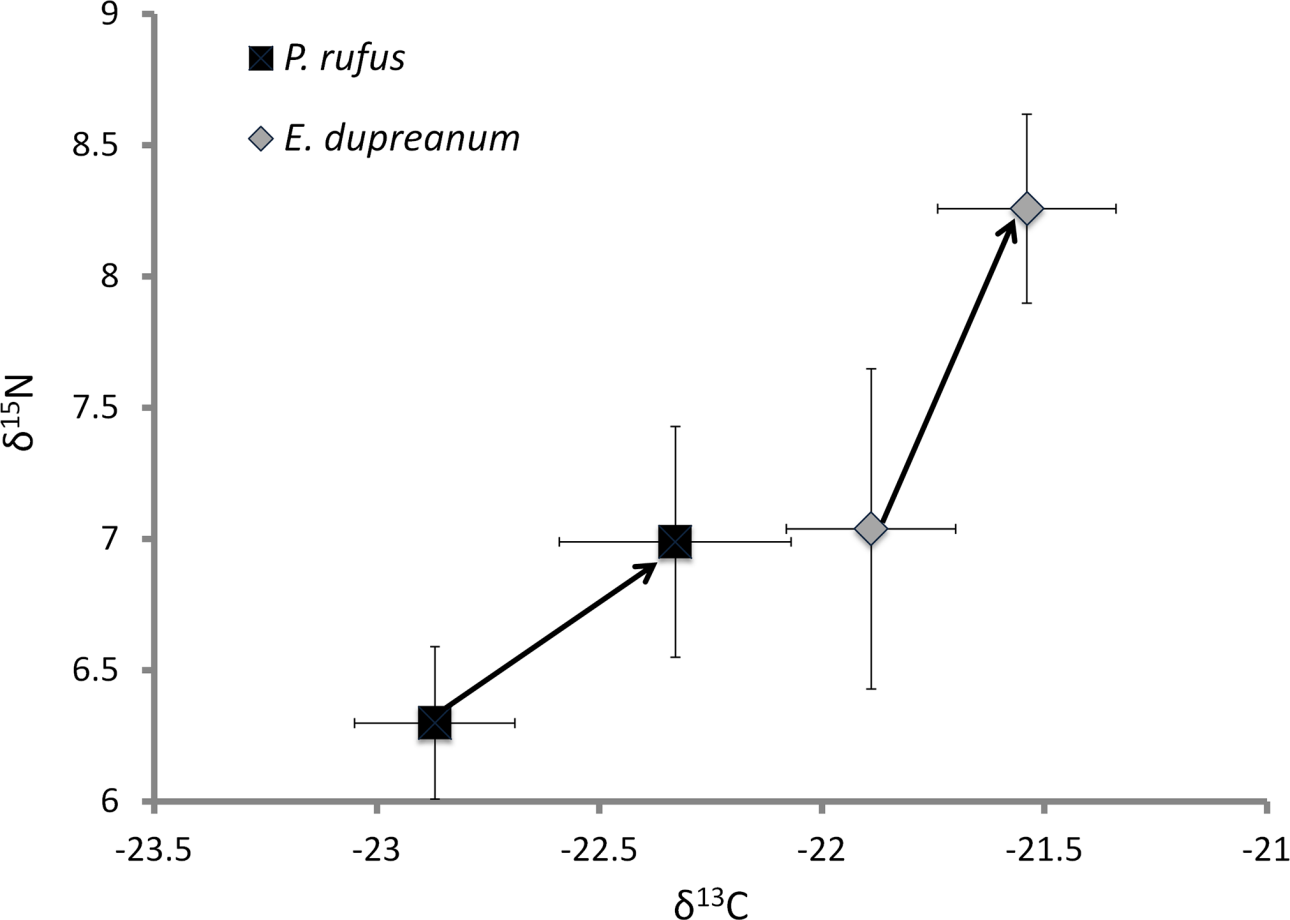
Dietary differences in *P*. *rufus* and *E*. *dupreanum* when caught alone or alongside each other. Differences in diet (δ^15^N and δ^13^C values) when *P*. *rufus* and *E*. *dupreanum* were caught at hunting sites alone (start of arrow) and when they were caught alongside each other (end of arrow). When caught in the presence of another species, both *P*. *rufus* (diamonds) and *E*. *dupreanum* (squares) significantly shifted in their δ^15^N and δ^13^C values. Hunting sites are replicates in this analysis, and the two species were caught alongside each other at three hunting sites (n = 36 *P*. *rufus* individuals and n = 24 *E*. *dupreanum* individuals), *E*. *dupreanum* was caught alone at a further three sites (n = 28 individuals) and *P*. *rufus* was caught alone at 11 hunting sites (n = 102 *P*. *rufus* individuals). Data show mean ± SD.

Second, at hunting sites where *P*. *rufus* was caught alongside *E*. *dupreanum*, δ^13^C values (Wilcoxon Test, Chi-square = 15.99, p < 0.0001; hunting sites as replicates) and δ^15^N values (Wilcoxon Test, Chi-square = 5.89, p = 0.0152) were higher than in areas where they were caught alone (n = 11 hunting sites where *P*. *rufus* was caught alone, n = 3 hunting sites where *P*. *rufus* was caught alongside *E*. *dupreanum*). Likewise, *E*. *dupreanum* had higher δ^13^C and δ^15^N values when caught alongside *P*. *rufus* than when caught alone (n = 3 hunting sites where *E*. *dupreanum* caught alongside *P*. *rufus*, n = 3 hunting sites were *E*. *dupreanum* caught alone, Wilcoxon Test, Chi-square = 7.28, p = 0.007 and Chi-square = 10.68, p = 0.0011, respectively; Fig 3). *R*. *madagascariensis* was always caught alongside *P*. *rufus*; it was not possible to determine whether it modified its resource use in the presence of other species.

### Regional Differences and Impact of Forest Cover and Annual Temperature Range on Diets

#### Regional changes in diet

In accordance with our hypothesis, both *P*. *rufus* and *E*. *dupreanum* diets (measured as Bray-Curtis similarity of δ^13^C and δ^15^N values) differed by hunting site (PERMANOVA, *P*. *rufus*, Pseudo-F = 5.41, df = 13, p = 0.001; *E*. *dupreanum*, Pseudo-F = 3.88, df = 5, p = 0.037; individuals as replicates).

#### Influence of forest cover on diet

There was some support for our hypothesis, as the diets of both *P*. *rufus* and *E*. *dupreanum* were affected by forest cover, albeit at different scales. After controlling for annual temperature range, the diet of *P*. *rufus* was best explained by forest cover at the largest spatial scale examined. Specifically, δ^13^C and δ^15^N values for *P*. *rufus* were best explained by a combination of the annual temperature range (average, annual temperature range during 1950–2000) and remnant forest cover within 30 km of their hunting site; the best model for δ^13^C also contained the temperature-forest cover interaction (Table 5). For δ^15^N, models with annual temperature range and different forest cover radii (5 and 15 km) also had some support. Further examination of the best models for *P*. *rufus* revealed that the influence of the forest cover-temperature interaction was strong for δ^13^C values (mixed effects model; forest cover within 30 km = 0.01, Std. Error = 0.02, p = 0.42; annual temperature range = -0.02, Std. Error = 0.01, p = 0.15; interaction = -0.004, Std. Error = 0.001, p = 0.012; model R^2^ = 0.41), but that the forest cover did not influence δ^15^N values (mixed effects model; forest cover within 30 km = -0.03, Std. Error = 0.02, p = 0.32; annual temperature range = -0.01, Std. Error = 0.02, p = 0.68; model R^2^ = 0.27). When these models were analyzed using hunting sites as replicates (n = 14 for *P*. *rufus*), δ^13^C and δ^15^N values were best explained by models with a single predictor variable. Models with the single variable of annual temperature range explained stable isotope variables the best, although models containing the single predictor of forest cover at all three radii also received some support (ΔAICc < ~2; Table 5).

**Table 5:** Results of model selection (ΔAICc < 2), with corrected Akaike’s information criterion (AICc) and Akaike weights (*w*_*i*_).

Fixed Effects	AICc	ΔAICc	*w*_*i*_
*P*. *rufus*– δ^13^C values (individuals as replicates, ‘hunting site’ as random effect)			
T + FC30 + T * FC30	355.58	0	0.45
*P*. *rufus–* δ^15^N values (individuals as replicates, ‘hunting site’ as random effect)			
T + FC30	506.71	0	0.36
T + FC5	508.18	1.47	0.17
T + FC30 + T * FC30	508.32	1.61	0.16
T + FC15	508.54	1.83	0.14
*P*. *rufus–* δ^13^C values (hunting sites as replicates, no random effects)			
T	36.39	0	0.28
FC15	37.10	0.71	0.20
FC30	37.86	1.47	0.14
FC5	37.91	1.52	0.13
T + FC30 + T * FC30	38.17	1.78	0.12
*P*. *rufus–* δ^15^N values (hunting sites as replicates, no random effects)			
T	39.03	0	0.35
FC30	39.79	0.77	0.24
FC5	40.91	1.87	0.14
*E*. *dupreanum–* δ^13^C values (hunting sites as replicates, no random effects)			
FC15	18.49	0	0.75
*E*. *dupreanum–* δ^15^N values (hunting sites as replicates, no random effects)			
FC5	27.76	0	0.40
T	28.26	0.50	0.31
FC30	29.28	1.52	0.19

Complete list of models tested with results can be found in S1 Table. The predictor variables included: T = annual temperature range; FC5 = forest cover within 5 km radius of hunting site; FC15 = forest cover within 15 km radius of hunting site; and FC30 = forest cover within 30 km radius of hunting site.

Annual temperature range was a poor predictor of δ^13^C values in *E*. *dupreanum*. The diet of *E*. *dupreanum* was correlated with forest cover, though at smaller spatial scales than *P*. *rufus*. *E*. *dupreanum* diets were best explained by forest cover within 15 km (δ^13^C values; Table 5) or 5 km (δ^15^N values) of the hunting site. For δ^15^N, alternate models with larger forest cover radii or annual temperature range also had some support. Further examination of the best models for *E*. *dupreanum* indicated forest cover had a significant influence on δ^13^C values (Regression; forest cover within 15 km, p = 0.006; model R^2^ = 0.88) but not on δ^15^N values (Regression; forest cover within 5 km, p = 0.12; model R^2^ = 0.50). Sample size was too low to analyze the impacts of annual temperature range and forest cover on *R*. *madagascariensis* diet.

## Discussion

### Diets and Diet Breadth of Fruit Bats in Madagascar

The three species rely on similar plant resources and have overlapping ranges [32,33]. Therefore, we expected the bats to show evidence of trophic niche differentiation due to resource competition; this was supported by our results. While the species differed in their diets, there was overlap in their diets, and (in accordance with Dammhahn & Goodman [14]) the differences among the three species in mean δ^13^C and δ^15^N values were not large in magnitude, suggesting relatively little separation in their diets. For example, mean δ^13^C values for the three species differed by only ~1.1‰ (range: -21.2 to -22.29‰), whereas a dry season study of fruit bats in South America found a difference of 6.8‰ (range: -21.5 to -28.3‰ [23]). The small species-level differences in stable isotope values cannot be explained by distinct and highly specialized diets in the three species; our results suggest substantial dietary overlap (Fig 2), in line with prior studies [32,33]. Another possible reason for dietary overlap is a decrease in resource availability over the dry season in seasonal habitats [13]. Any such seasonal restriction in diet composition, however, would only be captured partially in our results (the timing of our data collection and the turnover rate of hair samples [16] means that the results encompass both wet and dry seasons). Thus, we hypothesize that other causes of limited fruit availability, such as the replacement of native forests that provide diverse food resources with human-dominated landscapes that provide less diverse food resources [29,31], could be driving the diet overlap we observed.

*P*. *rufus* had a larger diet breadth than the two other species. This accords with prior studies, which have recorded *P*. *rufus* consuming a larger number of plant species [12,29] than the other two species [38,39]. It also suggests that *P*. *rufus* may more commonly use native habitats, where food sources are more diverse than in degraded habitats or orchards.

In terms of vertical stratification, *P*. *rufus* had lower δ^13^C values than *E*. *dupreanum*, which could indicate that *P*. *rufus* feeds higher in the forest canopy than *E*. *dupreanum* [20,23]. Though absolute differences in the stable isotope values were small, even small differences in δ^13^C values can indicate differences in foraging height of a half-dozen meters or more [23]. This is also consistent with observations that when the two species forage simultaneously in kapok trees (*Ceiba pentandra*), *P*. *rufus* forages in higher branches than *E*. *dupreanum* [33]. In regards to δ^15^N values, the higher δ^15^N values of *E*. *dupreanum* may indicate that it feeds on more commercially grown fruits [21] or in areas that are non-forested and dominated by grasslands and drought-resistant plants [62]. This interpretation accords well with observations that *E*. *dupreanum* more readily uses degraded habitats than *P*. *rufus* [32].

The diets of both bats shifted similarly–toward higher δ^13^C and δ^15^N values–when captured together. Specifically, the diet of *P*. *rufus* when the two species co-occurred coincided with the diet of *E*. *dupreanum* in the absence of other bats. Further, with *P*. *rufus* present, the diet of *E*. *dupreanum* shifted to even higher δ^13^C and δ^15^N values (Fig 3). Although no clear conclusions can be drawn without baseline data, these results may point to a scenario in which the larger and likely more competitively-dominant *P*. *rufus* is able to exclude *E*. *dupreanum* from its primary foraging areas in the higher canopy levels of the primary forest, leaving *E*. *dupreanum* to forage at a lower canopy height (higher δ^13^C values) and in more degraded areas including savannahs and agricultural areas (higher δ^15^N values). In this scenario, when primary forest is unavailable, *P*. *rufus* may shift its foraging to more degraded habitats (higher δ^15^N values) that lack high canopies (higher δ^13^C values), displacing *E*. *dupreanum* to even more degraded habitats (Fig 3). A closer examination of the hunting sites also supports this idea; sites where the two species were caught together had less remnant habitat (9 ± 5% within 15 km and 7 ± 4% within 30 km) than those where they were caught separately (20 ± 11% and 16 ± 11%, respectively).

Our analysis of diet shifts assumed that data on bat captures could be used to infer co-occurrence. However there was a difference between the time frame of the foraging behavior (the shared use of foraging sites was determined for the night captured) and the turnover time for stable isotopes in hair (several months [16]). Thus, while spatial patterns of bat captures are likely to correlate with co-occurrence over time, bias could be introduced if patterns of bat occurrence on the night of capture were not representative of typical patterns of occurrence on a months-long time frame.

We were unable to examine whether *R*. *madagascariensis* changed its foraging behavior in the presence of another species. However, it is likely that the diet overlaps with the other species (Fig 2, [14]). This overlap could be maintained through the use of different foraging behavior by *R*. *madagascariensis*. Unlike the other two species, *R*. *madagascariensis* hovers and removes fruit in flight before bringing it to a nearby feeding roost [13]. This may enable it to access resources that would be inaccessible to the other two species, and thus avoid competition with them even when foraging on the same fruit trees.

### Impact of Forest Cover on Bat Diets

The loss of forest cover may impact *P*. *rufus* at larger spatial scales (30 km) than *E*. *dupreanum* (5–15 km). These data fit well with previous findings that *P*. *rufus* is able to forage across a wider range than *E*. *dupreanum* [18]. Because *P*. *rufus* has access to a wider geographic range [18], it may have more options to choose from in terms of foraging. This increased access may be reflected in the lower δ^15^N values of *P*. *rufus* (compared to *E*. *dupreanum*). These δ^15^N values increase in drought-resistant plants and in agricultural areas [20] where primary forests have been heavily degraded. In such locations, *E*. *dupreanum* may have few options but to forage in degraded habitats (because it cannot travel as far as *P*. *rufus* [18]), whereas *P*. *rufus*, with its greater flight radius, has flexibility to visit farther, more intact habitats. Our dataset was not large enough to analyze the impact of deforestation on *R*. *madagascariensis*. However, their short foraging distances relative to the other two Malagasy fruit bat species [37] imply that *R*. *madagascariensis* may be responsive to habitat change across smaller spatial scales.

The impact of the interaction between forest cover and annual temperature range on the δ^13^C values of *P*. *rufus* was greater than the impact of forest cover alone. This suggests that *P*. *rufus* is vulnerable to the combined impacts of annual temperature range–which may change the availability of fruits and resources–and a decrease in remnant habitat. It may be that *P*. *rufus* has the ability to overcome the impacts of annual temperature range or the impacts of forest loss individually, but not the combination of the two.

There are several considerations and factors that were not accounted for in our statistical analysis, but which could have affected our interpretation of the data. First, stable isotope values can be affected by changes in the dominant forest type. However, given that all hunting sites in our study had the same dominant forest type (Table 3), we hypothesize that the dietary changes observed are due primarily to a loss in forest cover, not a change in forest type or geographic variation. Second, in the spatial analysis of forest cover, we used hunting sites as centroids, which could introduce bias if hunting sites were actually near the edge of a bat’s nightly foraging range and if forest cover differed substantially outside the foraging range. However, the possibility for such bias seems limited since hunting sites for bats in Madagascar are often at or near roost sites [30,32]. Finally, although each of the bat species appear tolerant of degraded areas [31], *P*. *rufus* dietary breadth may decrease substantially in degraded areas. As such additional research is needed to understand to what extent bats are negatively affected by the restriction of diets associated with loss of forest and if so, how much forest loss the bats can withstand.

### Conservation Implications

Our data suggest that the three species had broadly overlapping diets but that there were differences in the diets of *P*. *rufus* and *E*. *dupreanum*. These diets shifted when the species co-occurred, suggesting resource partitioning across habitats and vertical strata within the canopy to avoid competition, with *P*. *rufus* perhaps foraging more in native forests and at a higher forest strata compared to *E*. *dupreanum*. Diets changed as the percent of forest cover decreased, though at a larger spatial scale in *P*. *rufus* than in *E*. *dupreanum*. These results suggest that the species exhibit differing responses to habitat change, highlight the threats fruit bats face from habitat change, and clarify the spatial scales at which conservation efforts could be implemented. Specifically, our data provide evidence that the scale at which conservation should be implemented may differ for the three species. For example, based on the finding of the significant effect of forest cover on a 30 km scale, a conservation strategy aimed at protecting *P*. *rufus* might include a focus on protecting sufficient patches of primary forest within ~30 km of its roots to maintain sufficient foraging resources. Conversely, programs aimed at protecting *R*. *madagascariensis* and *E*. *dupreanum*, could focus on maintaining foraging resources at smaller (5–15 km) scales. It is important to note that such an emphasis on maintaining foraging habitat is important but not sufficient for conserving fruit bats in Madagascar; for example, hunting at roost locations is a primary threat facing these species [29,31]. Promisingly, our sampling of bat hair via the wild meat trade confirms the presence of bats near known roost sites [32] and suggests the presence of previously unknown roosts (e.g., near Andriba and Antsiafabositra).

## Supporting Information

S1 Appendix Stable isotope values (δ^15^N and δ^13^C values) for each sample collected. Includes species identification, city of purchase, location hunted (including GPS coordinates), % of forest cover (within 5km, 15km, and 30km radius), city population, annual precipitation, annual temperature, temperature seasonality, and precipitation seasonality.
(XLSX)

S1 Fig Comparison of the areas of the standard ellipses for each species. Calculated using a Bayesian approach (10^5^ posterior draws).
(JPEG)

S1 Table Complete results of model selection, with corrected Akaike’s information criterion (AICc), ΔAICc, and Akaike weights (*w*_*i*_). (DOCX)
