# Supplementary figures and images for: Using Stable Isotopes to Infer the Impacts of Habitat Change on the Diets and Vertical Stratification of Frugivorous Bats in Madagascar
Source: PLoS One. 2016 Apr 20;11(4):e0153192. doi: 10.1371/journal.pone.0153192 (PMC4838311; doi:10.1371/journal.pone.0153192)

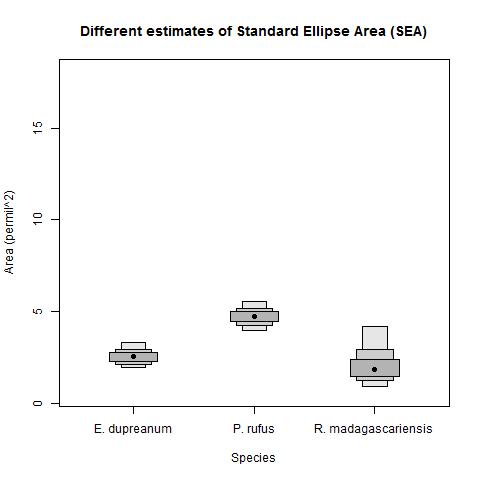

Supplement: S1 Fig — Calculated using a Bayesian approach (105 posterior draws). (JPEG) [file pone.0153192.s002.jpeg]
