# Supplementary material for: Using Stable Isotopes to Infer the Impacts of Habitat Change on the Diets and Vertical Stratification of Frugivorous Bats in Madagascar
Source: PLoS One. 2016 Apr 20;11(4):e0153192. doi: 10.1371/journal.pone.0153192 (PMC4838311; doi:10.1371/journal.pone.0153192)
Supplement: S1 Table — (DOCX) [file pone.0153192.s003.docx]

**Table S1. Results of model selection, with corrected Akaike’s information criterion (AICc), ΔAICc, and Akaike weights (*w_i_*).**

| **Fixed Effects** | **AICc** | **ΔAICc** | ***w_i_*** |
| --- | --- | --- | --- |
| *P. rufus* – δ^13^C values (individuals as replicates, ‘hunting site’ as random effect) | | | |
| T + FC30 + T * FC30 | 355.58 | 0 | 0.45 |
| T + FC30 | 357.95 | 2.38 | 0.14 |
| T + FC15 | 358.11 | 2.53 | 0.13 |
| T + FC5 | 358.75 | 3.17 | 0.09 |
| T + FC15 + T * FC15 | 358.87 | 3.30 | 0.09 |
| T | 359.09 | 3.51 | 0.08 |
| T + FC5 + T * FC5 | 362.56 | 6.98 | 0.01 |
| FC30 | 366.15 | 10.57 | 0.00 |
| FC15 | 366.25 | 10.68 | 0.00 |
| None (intercept only) | 367.07 | 11.49 | 0.00 |
| FC5 | 367.07 | 11.49 | 0.00 |
| *P. rufus* – δ^15^N values (individuals as replicates, ‘hunting site’ as random effect) | | | |
| T + FC30 | 506.71 | 0 | 0.36 |
| T + FC5 | 508.18 | 1.47 | 0.17 |
| T + FC30 + T * FC30 | 508.32 | 1.61 | 0.16 |
| T + FC15 | 508.54 | 1.83 | 0.14 |
| T | 509.29 | 2.58 | 0.10 |
| T + FC15 + T * FC15 | 511.68 | 4.97 | 0.03 |
| T + FC5 + T * FC5 | 511.80 | 5.09 | 0.03 |
| FC30 | 524.06 | 17.35 | 0.00 |
| FC5 | 526.00 | 19.29 | 0.00 |
| FC15 | 526.29 | 19.58 | 0.00 |
| None (intercept only) | 526.89 | 20.18 | 0.00 |
| *P. rufus* – δ^13^C values (hunting sites as replicates, no random effects) | | | |
| T | 36.39 | 0 | 0.28 |
| FC15 | 37.10 | 0.71 | 0.20 |
| FC30 | 37.86 | 1.47 | 0.14 |
| FC5 | 37.91 | 1.52 | 0.13 |
| T + FC30 + T * FC30 | 38.17 | 1.78 | 0.12 |
| T + FC15 | 40.11 | 3.73 | 0.04 |
| T + FC15 + T * FC15 | 40.46 | 4.07 | 0.04 |
| T + FC5 | 41.04 | 4.65 | 0.03 |
| T + FC30 | 41.09 | 4.70 | 0.03 |
| T + FC5 + T * FC5 | 45.07 | 8.68 | 0.00 |
| *P. rufus* – δ^15^N values (hunting sites as replicates, no random effects) | | | |
| T | 39.03 | 0 | 0.35 |
| FC30 | 39.79 | 0.77 | 0.24 |
| FC5 | 40.91 | 1.87 | 0.14 |
| FC15 | 41.16 | 2.13 | 0.12 |
| T + FC30 | 42.66 | 3.64 | 0.06 |
| T + FC5 | 43.48 | 4.46 | 0.04 |
| T + FC15 | 43.72 | 4.70 | 0.03 |
| T + FC30 + T * FC30 | 45.63 | 6.60 | 0.01 |
| T + FC15 + T * FC15 | 46.79 | 7.76 | 0.01 |
| T + FC5 + T * FC5 | 47.02 | 7.99 | 0.01 |
| *E. dupreanum* – δ^13^C values (hunting sites as replicates, no random effects) | | | |
| FC15 | 18.49 | 0 | 0.75 |
| FC30 | 21.03 | 2.54 | 0.21 |
| T | 24.73 | 6.24 | 0.03 |
| FC5 | 30.96 | 12.47 | 0.00 |
| *E. dupreanum* – δ^15^N values (hunting sites as replicates, no random effects) | | | |
| FC5 | 27.76 | 0 | 0.40 |
| T | 28.26 | 0.50 | 0.31 |
| FC30 | 29.28 | 1.52 | 0.19 |
| FC15 | 30.46 | 2.70 | 0.10 |

The predictor variables included: T = annual temperature range; FC5 = forest cover within 5 km radius of hunting site; FC15 = forest cover within 15 km radius of hunting site; and FC30 = forest cover within 30 km radius of hunting site.
